# Mechanisms of Kale (*Brassica oleracea* var. *acephala*) Tolerance to Individual and Combined Stresses of Drought and Elevated Temperature

**DOI:** 10.3390/ijms231911494

**Published:** 2022-09-29

**Authors:** Nataša Bauer, Mirta Tkalec, Nikola Major, Ana Talanga Vasari, Mirta Tokić, Sandra Vitko, Dean Ban, Smiljana Goreta Ban, Branka Salopek-Sondi

**Affiliations:** 1 Department for Molecular Biology, Faculty of Science, University of Zagreb, 10000 Zagreb, Croatia; 2 Institute of Agriculture and Tourism, 52440 Poreč, Croatia; 3 Centre of Excellence for Biodiversity and Molecular Plant Breeding, 10000 Zagreb, Croatia; 4 Laboratory for Chemical Biology, Department for Molecular Biology, Ruđer Bošković Institute, 10000 Zagreb, Croatia

**Keywords:** growth performance, heat shock proteins, photosynthetic efficiency, stress markers, *NAC*, *HSFs*, *DREB*

## Abstract

Rising temperatures and pronounced drought are significantly affecting biodiversity worldwide and reducing yields and quality of Brassica crops. To elucidate the mechanisms of tolerance, 33 kale accessions (*B. oleracea* var. *acephala*) were evaluated for individual (osmotic and elevated temperature stress) and combined stress (osmotic + temperature). Using root growth, biomass and proline content as reliable markers, accessions were evaluated for stress responses. Four representatives were selected for further investigation (photosynthetic performance, biochemical markers, sugar content, specialized metabolites, transcription level of transcription factors *NAC*, *HSF*, *DREB* and expression of heat shock proteins HSP70 and HSP90): very sensitive (392), moderately sensitive (395), tolerant (404) and most tolerant (411). Accessions more tolerant to stress conditions were characterized by higher basal content of proline, total sugars, glucosinolates and higher transcription of *NAC* and *DREB*. Under all stress conditions, 392 was characterized by a significant decrease in biomass, root growth, photosynthesis performance, fructan content, especially under osmotic and combined stress, a significant increase in *HSF* transcription and HSP accumulation under temperature stress and a significant decrease in *NAC* transcription under all stresses. The most tolerant accession under all applied stresses, 411 showed the least changes in all analyzed parameters compared with the other accessions.

## 1. Introduction

The Brassicaceae family is cultivated worldwide and includes many economically important species. An important agricultural genus in the Brassicaceae family is *Brassica*, which includes oilseeds (canola, mustard) and vegetables (broccoli, cabbage, cauliflower, kale, kohlrabi) that have long been used for agriculture on all continents [1]. Brassica vegetables have attracted public and scientific attention because of their health potential due to their richness in “healthy phytochemicals” (carotenoids, phenols, glucosinolates, vitamins) and their great adaptability to climatic conditions that allow for their cultivation all over the world. Various epidemiological and meta-analyses suggest that consumption of *Brassica* vegetables plays a preventive role against a number of chronic diseases due to its antioxidant, antimicrobial and anticancer effects and these findings allow for the recognition of *Brassica* vegetables as a functional food [2,3]. One cruciferous vegetable that frequently appears on lists of ‘healthiest foods’ or ‘superfoods’ is kale (*Brassica oleracea* var. *acephala)*, a leafy, non-headed cabbage. Although kale has been cultivated for several centuries and is found in many traditional dishes, especially in the Mediterranean region, it became very popular in the United States after 2010 [3].

However, the cultivation of brassicas, like that of other crops, is strongly affected by climate change. Increasing temperatures and decreasing precipitation are causing abiotic stress in plants, which can significantly affect biodiversity worldwide and reduce crop yields and quality of agriculturally important *Brassica* species, which, in turn, affects food chain resilience. Approximately 40% of the world’s land area is affected by drought. Average surface temperatures have already increased by about 1 °C from 1990 to the present and global average surface temperatures are expected to increase by 2.6 to 4.8 °C by the end of the century, resulting in a warmer and drier climate [4]. Drought and heat stress usually occur together and are the major obstacles to crop production [5]. The problem is particularly severe in Mediterranean, semi-arid and arid regions and is expected to worsen in the future [6]. High temperatures affect plant growth and development of Brassica species and lead to yield losses. For example, a yield reduction of 17% was observed in Brassica plants exposed to a temperature increase of 1 °C [7].

Plant response to stress caused by abiotic factors is a complex trait regulated by many factors (gene expression, plant hormones, antioxidants, osmoprotectants, etc.) [8]. Transcriptional regulation of stress-responsive genes is an essential step in determining the mechanisms of tolerance to abiotic stress. Transcription factors (TFs), as major regulators of gene expression, may be important targets for the development of crops with enhanced abiotic stress tolerance. Several transcription factors, such as heat shock transcription factors (HSFs), dehydration-responsive element binding protein (DREB) and NAC (NAM, ATAF and CUC domain) are reported as key regulators under stress conditions that control the transcription of stress-related genes and the synthesis of stress-responsive proteins that ensure plant survival under stress conditions [9,10].

Our recent comparative studies of *Brassica* species have shown that kale is more tolerant to salinity and drought compared to Chinese cabbage and white cabbage [11,12]. Furthermore, kale shows good tolerance to extreme temperature fluctuations [3]. Due to its good tolerance to abiotic stresses caused by climate change, kale has become very popular among farmers in recent decades. Kale production significantly increased in the US, from 3994 to 6256 harvested acres, in the period from 2007 to 2012, respectively [13]. In the last few decades, morphological, agronomical, genetical and phytochemical characteristics of local kale varieties have been considered and investigated in many countries [3].

Kale is commonly grown along the coastal region of Croatia on small family farms and with low commercial importance. Since each farmer is preserving their own seeds, a significant morphological diversity among kale populations is present in the region [14]. There is a possibility that more tolerant accessions to abiotic stress are present in the area and, therefore, could provide important genetic resources for selection of the cultivars more adapted to climate changes.

Although many studies have been focused to shed light on tolerance mechanisms [8], there is still a large gap in understanding the complex network of multiple processes involved in the mechanisms of stress tolerance. Therefore, research on the mechanisms of tolerance is of great importance from an ecological and agronomic point of view [15].

The main objective of this work was to investigate the tolerance of 33 local kale accessions (*B. oleracea* var. *acephala*) to individual (osmotic and heat stress) and combined stress (osmotic + heat). Based on growth performance (root growth and biomass) and proline level as a reliable stress marker, the most tolerant and the most sensitive accessions were selected to shed light on the mechanisms of abiotic stress tolerance.

## 2. Results

### 2.1. Screening of Kale Accessions to Abiotic Stresses

**Figure 1:**
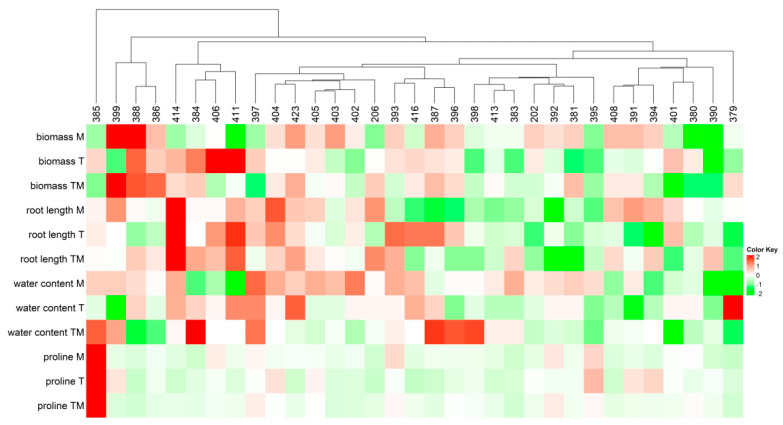
Heatmap and cluster analysis of biomass, root length, water and proline content in 33 *Brassica oleracea* var. *acephala* accessions. Abbreviations M, T and TM represent plants treated with mannitol, elevated temperature and a combination of mannitol and elevated temperature, respectively. The relative value for each parameter and accession is normalized to the respective control and is represented by color intensity, with red indicating higher values and green indicating lower values.

To shed light on the mechanisms of abiotic stress tolerance, 33 kale accessions (Table 1) were analyzed for elevated temperature (T), osmotic stress (M) and combined stress (elevated temperature + osmotic stress; TM) (Figure 1A, Table S1).

As can be seen, kale accessions were grouped based on their growth performance (biomass accumulation and root growth), water content and proline content as reliable stress markers (Figure 1). The more tolerant accessions were characterized by higher (more intense red color) biomass production, greater root length, higher water content and lower (more intense green color) proline content under stress conditions. The most severe growth inhibition (biomass accumulation and root growth), reduction in water content and increase in proline content were associated with osmotic stress (M) and combined stress (TM), while temperature stress alone (T) caused less noticeable changes compared to controls (C) (Table S1).

### 2.2. Selection of Tolerant and Sensitive Accessions

**Figure 2:**
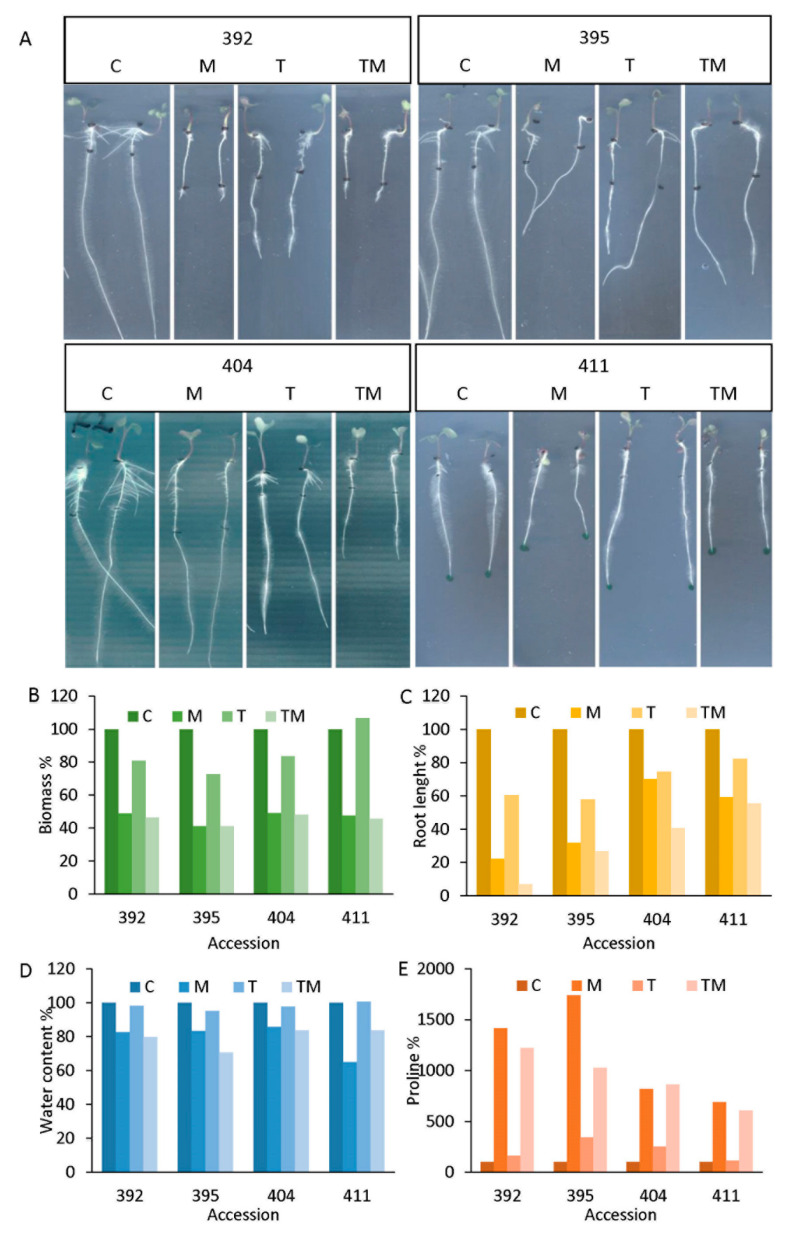
Accessions 392, 395, 404 and 411 selected for further analysis: (**A**) photographs of kale seedlings from accessions 392, 395, 404 and 411 under stress conditions (abbreviations M, T and TM represent plants treated with mannitol, elevated temperature and a combination of mannitol and elevated temperature, respectively) compared to the corresponding controls (**C**). Stress markers as % of the corresponding controls (C = 100%): (**B**) biomass, (**C**) root length, (**D**) water content and (**E**) proline level for accessions selected as sensitive (392 and 395) and tolerant (404 and 411) to the stresses studied. Raw data are presented in Table S1.

Based on growth parameters (biomass accumulation and root length), water content and proline level, four kale accessions with different stress responses were selected: 392, 395, 404 and 411 (Figure 2). These accessions were selected as representative models for a group of accessions that responded similarly to the stress factors studied. Accessions 392 and 395 were considered to be more sensitive representatives among examined accessions, while accessions 404 and 411 were considered to be more tolerant representatives of the 33 studied accessions to the applied stresses compared to the corresponding controls (Figure 2A). As can be seen, root length and proline level were the most distinctive parameters between tolerant and sensitive accessions (Figure 2B). Root lengths reached 22%, 61% and 7% of length in accession 392 and 32%, 58% and 27% of length in accession 395 under osmotic, temperature and combined stress, respectively, compared to the corresponding controls. On the other hand, in more tolerant accessions, root length reached 70%, 75% and 41% of length in accession 404 and 59%, 82% and 56% of length in accession 411 under osmotic, temperature and combined stress, respectively, compared to the corresponding controls. Proline content was significantly increased under stress conditions, but more prominently in the sensitive accessions (up to 14.2-fold in accession 392 under osmotic stress and up to 17.4-fold in accession 395 under osmotic stress compared to the controls) than in the more tolerant ones (up to 8.6-fold in accession 404 under combined stress and 6.9-fold in accession 411 under osmotic stress compared with controls) (Table S1).

Interestingly, the basal level of proline under control conditions was lower in the sensitive accessions (0.8 μg mg^−1^ DW in 392 and 0.6 μg mg^−1^ DW in 395) than in the more tolerant accessions (2.4 μg mg^−1^ DW in 404 and 2.7 μg mg^−1^ DW in 411) (Table S1).

Water content was most reduced in seedlings treated with mannitol and combined stress in 392 (16% and 18%, respectively) and 395 (15% and 27%, respectively) accessions. A similar decrease in water content was seen in 404 under mannitol and combined stress (13% and 15%, respectively). Temperature stress caused a 2–4% decrease in water content for accessions 392, 395 and 404. Accession 411 performed well under temperature stress and showed no loss of water content under temperature stress compared to the control. However, in this accession, mannitol caused a significant decrease in water content (30%), while the combined stress resulted in a 15% decrease in water content compared to the control.

### 2.3. Selection of Tolerant and Sensitive Accessions

**Figure 3:**
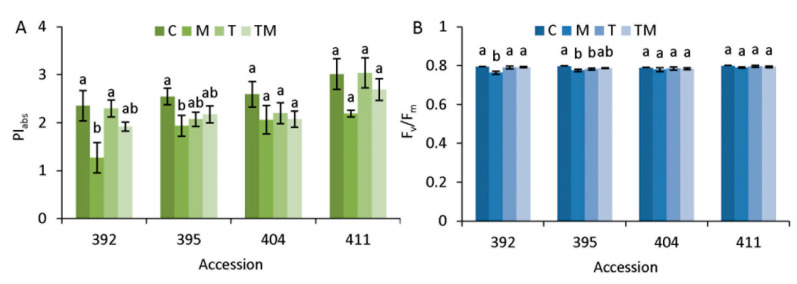
Photosynthetic parameters (**A**) PI_abs_ and (**B**) F_v_/F_m_ measured in the selected kale accessions: 392, 395, 404 and 411. Abbreviations M, T and TM represent plants treated with mannitol, elevated temperature and a combination of mannitol and elevated temperature, respectively, compared to the corresponding controls, C. Data are average values of n = 5 ± SE. Different letters denote significant difference between treatments of each accession (*p* < 0.05, one-way ANOVA, LSD test).

Photosynthetic performance parameters PI_abs_ and F_v_/F_m_ were chosen to present the influence of abiotic stresses on the status of the photosynthetic apparatus of selected kale accessions (Figure 3).

The PI_abs_ parameter was more sensitive to the applied abiotic stressors than the F_v_/F_m_ parameter. As can be seen, osmotic stress (M) caused a significant decrease in PI_abs_ compared to the control in accessions 392 (1.9-fold) and 395 (1.3-fold) (Figure 3A). However, this was not observed in accessions 404 and 411. Combined stress (TM) resulted in an approximately 1.2-fold decrease in PI_abs_ in all accessions compared with the corresponding control, but this was not statistically significant. Temperature stress (T) did not result in a significant change in PI_abs_ in any of the accessions tested. Compared to the control, the F_v_/F_m_ parameter changed significantly in accessions 392 and 395 under osmotic stress and in 395 also under temperature stress.

### 2.4. Stress Markers

**Figure 4:**
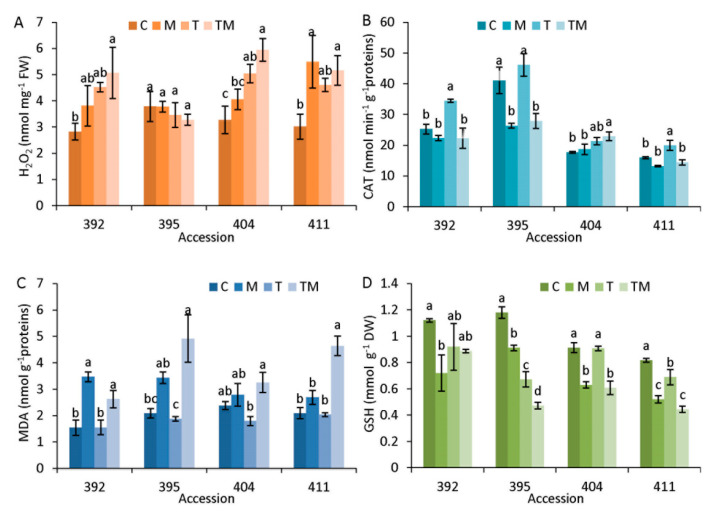
Oxidative stress markers (**A**) hydrogen peroxide (H_2_O_2_), (**B**) catalase (CAT) activity, (**C**) level of lipid peroxidation (MDA) and (**D**) reduced glutathione (GSH) content measured in the selected kale accessions: 392, 395, 404, 411. Abbreviations M, T and TM represent plants treated with mannitol, elevated temperature and a combination of mannitol and elevated temperature, respectively, compared to the corresponding controls, C. Data are average values of *n* = 3–5 ± SE. Different letters denote significant difference between treatments of each accession (*p* < 0.05, one-way ANOVA, LSD test).

To investigate the oxidative stress level caused by the application of mannitol, increased temperature and combined stress, the hydrogen peroxide level (H_2_O_2_), catalase activity (CAT), lipid peroxidation level (MDA) and reduced glutathione level (GSH) were analyzed (Figure 4).

As can be seen, H_2_O_2_ content was differently changed in examined accessions under diverse stress treatments (Figure 4A). H_2_O_2_ was significantly increased in 392 accessions only under combined stress compared to the corresponding control. It was significantly increased in 404 accessions under temperature and combined stress and in 411 accessions under osmotic and combined stress compared with the control (Figure 4A).

Content of H_2_O_2_ was not changed in the 395 accession under stress conditions. Interestingly, the control sample of 395 accession showed higher CAT activity compared to the controls of the other accessions, which decreased (Figure 4B). CAT activity increased it significantly under osmotic and combined stress (significantly, in accessions 392, 404 and 411 under combined or temperature stress, respectively, compared with controls). MDA content (Figure 4C) was significantly increased under osmotic and combined stress in 392 accession and under combined stress in accessions 395 and 411 compared with their corresponding controls. GSH content was higher in accessions 392 and 395 under control conditions than in accessions 404 and 411 (Figure 4D). A tendency for GSH content to decrease was observed in all accessions under the applied stress conditions. In accessions 395 and 411, GSH content was significantly decreased under all stress conditions compared to the control. Additionally, it was significantly decreased under osmotic and combined stress in the 404 accession and only under osmotic stress in the 392 accession compared to the control.

### 2.5. Specialized Metabolites and Antioxidant Activity

**Figure 5:**
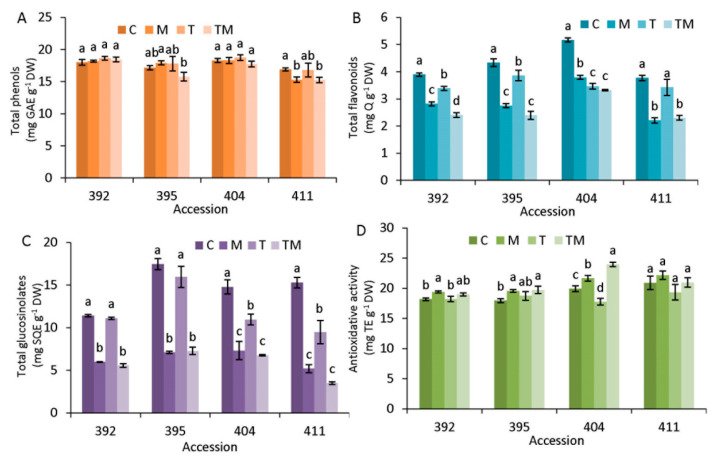
(**A**) Total phenols, (**B**) total flavonoids, (**C**) total glucosinolates and (**D**) antioxidative activity measured by DPPH in accessions 392, 395, 404 and 411 under osmotic stress, M, temperature stress, T, and combined stress, TM, compared to the control, C. Data are average of *n* = 3 ± SE. Different letters denote significant difference between treatments of each accession (*p* < 0.05, one-way ANOVA, LSD test).

Specialized metabolites: total phenols, total flavonoids and total glucosinolates were measured spectrophotometrically (Figure 5).

As shown, total phenols (Figure 5A) were decreased significantly only in the 411 accession under osmotic and combined stress compared with the control. Flavonoids (Figure 5B) decreased under all stress conditions compared with the control, except for temperature stress in accession 411 and the decrease was more pronounced under osmotic and combined stress. Accessions 395, 404 and 411 had higher total glucosinolate content than 392 under control conditions. Compared to the control, a significant decrease in glucosinolates was observed in all accessions under osmotic and combined stress. Moreover, accessions 404 and 411 showed a significant decrease in total glucosinolates under temperature stress. Antioxidant activity measured by the DPPH method did not show significant changes in the 411 accession, regardless of the stress applied. However, it was significantly increased compared to control in accessions 392, 395 and 404 under osmotic and combined stress.

### 2.6. Sugar Analysis

**Figure 6:**
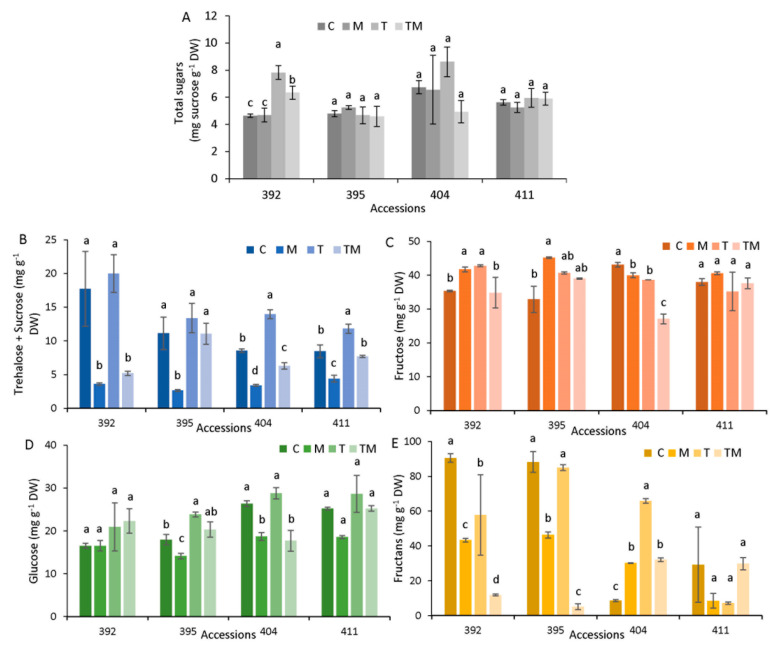
Sugar analysis in accessions 392, 395, 404 and 411 under stress conditions (M—mannitol, T—elevated temperature, TM—combined stress, C—control): (**A**) total sugars measured by spectrophotometry; (**B**) trehalose + sucrose, T+S, (**C**) fructose, F, (**D**) glucose, G and (**E**) fructans, FS, measured by HPLC. Data are average value ± SE, *n*= 3. Different letters denote significant difference between treatments of each accession (*p* < 0.05, one-way ANOVA, LSD test).

Total sugars were measured by spectrophotometry (Figure 6A). Control samples of accessions 392 and 395 had lower values of total sugars (4.6 and 4.8 mg g^−1^ DW, respectively), while accessions 404 and 411 had a higher level of total sugars (6.7 and 5.6 mg g^−1^ DW, respectively). The level of total sugars increased significantly under temperature (1.7-fold) and combined (1.4-fold) stress only in the 392 accession. Selected sugars, trehalose, sucrose, fructose and total fructans, were measured by HPLC. Under our chromatography conditions, trehalose and sucrose peaks overlapped; thus, we presented both sugars as a joint value (T + S). As can be seen in Figure 6B, osmotic stress decreased T+S significantly compared to corresponding controls in all accessions. Temperature stress did not change T+S level in accessions 392 and 395, while it caused a significant increase in T+S level in accessions 404 and 411. Fructose content (F) was increased in accessions 392 and 395, mostly under osmotic stress (Figure 6C).

In the 404 accession, fructose content significantly decreased in all applied treatments, while it was unchanged in the 411 accession. Glucose content (G) was not changed in the 392 accession under stress conditions, while in accessions 395 and 404, it decreased under osmotic and combined stress (Figure 6D). Fructans were present at a high level in accessions 392 and 395 under control conditions and decreased significantly under osmotic and combined stress, while elevated temperature did not significantly change fructan level in 395 (Figure 6E). On the other hand, fructans increased significantly in the 404 accession under all stress conditions, particularly under high temperature compared to the control. In the 411 accession, fructans did not change under stress conditions compared to the corresponding control.

### 2.7. Gene Expression Analysis

**Figure 7:**
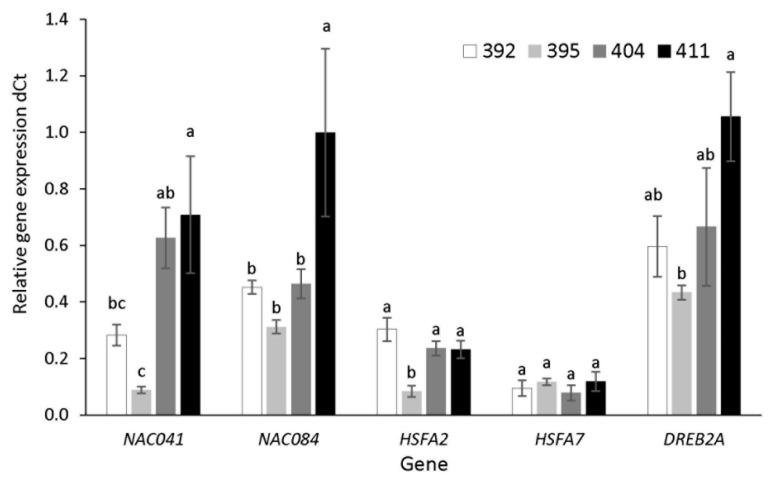
Basal relative gene expression of transcription factors *NAC041* and *NAC084*, *HSFA2*, *HSFA7* and *DREB2A* in accessions 392, 395, 404 and 411, under control condition measured by qPCR compared to referent genes (dCt). Data are average ± SE, *n*= 3. Different letters present significant difference between accessions for each individual gene (one-way ANOVA, LSD test).

Transcript level analysis was performed for heat shock transcription factors *HSFA2* and *HSFA7*, for *DREB2A* and transcription factors *NAC041* and *NAC084*. In control conditions, basal gene expression of transcription factors varied among accessions (Figure 7).

It is interesting that the most tolerant accession 411 had significantly higher basal gene expression of *NAC* factors and *DREB2A* compared to more sensitive accessions 392 and 395. Accession 395 showed the lowest basal gene expression of *NACs* and *HSFA2* and *DREB2A* transcription factors.

**Figure 8:**
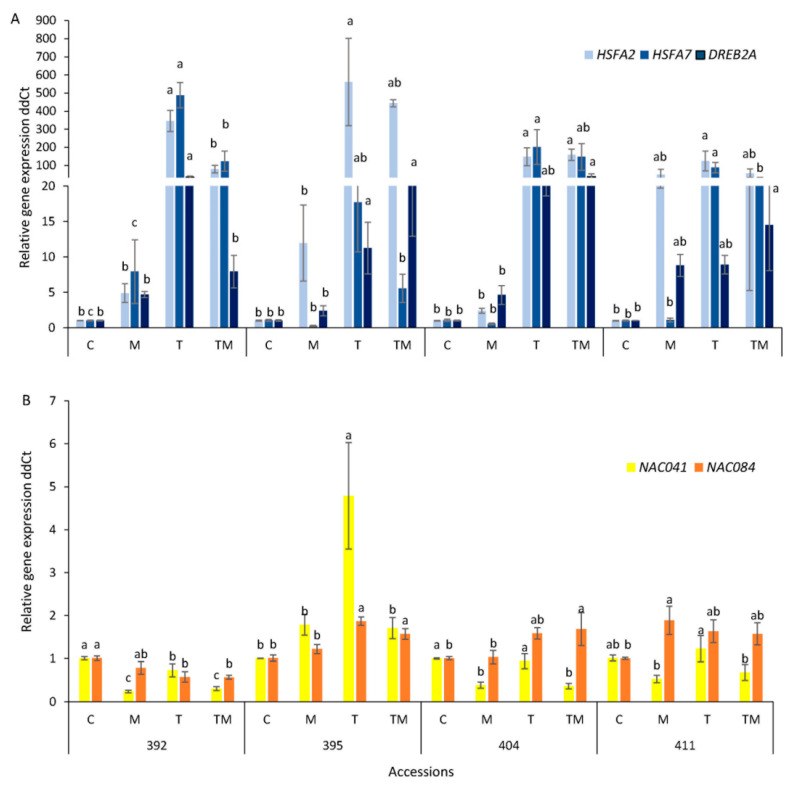
Relative gene expression of transcription factors (**A**) *HSFA2*, *HSFA7* and *DREB2A* and (**B**) *NAC041* and *NAC084*, in accessions 392, 395, 404 and 411, 24 h under control (C = 1) and osmotic stress, M, temperature stress, T and combined stress, TM, conditions measured by qPCR (ddCt). Gene expression was presented as fold change compared to the controls. Data are average ± SE, *n*= 3. Different letters present significant difference among treatments of individual accessions (one-way ANOVA, LSD test).

Changes in transcript level of selected genes under stress conditions are shown in Figure 8. As can be seen, a significant increase in *HSF* transcript level was observed in all accessions under temperature stress (Figure 8A, Table S3). The highest increase in *HSFA2* was observed in accession 395. Temperature stress induced increases of 345-, 560-, 148- and 124-fold in *HSFA2* expression in 392, 395, 404 and 411 accession, respectively, compared to the control. There was a tendency of increasing the expression of *HSFA2* in osmotic and combined stress, although the change was statistically significant only in accession 404 under combined stress. *HSFA7* expression was significantly induced under temperature stress in all accessions (488-, 17-, 202- and 88-fold in accessions 392, 395, 404 and 411, respectively) and in accession 392 under osmotic stress (8-fold), while osmotic stress reduced its expression in accessions 395 and 404, although changes were statistically non-significant.

*DREB2A* transcript was significantly increased under temperature stress in accession 392 (35-fold) while its transcription was increased in accessions 395 (11-fold), 404 (22-fold) and 411 (9-fold), although these increases were statistically non-significant (Figure 8A, Table S3). Combined stress caused a significant increase in *DREB2A* transcript level in 395, 404 and 411 (21-, 38- and 14-fold, respectively). Under mannitol stress, DREB2A showed an intention to increase in all accessions, although changes were statistically non-significant (Figure 8A, Table S3).

Considering NAC transcription factors, there was significant downregulation of *NAC041* and *NAC084* genes observed in the 392 accession under all stress conditions (Figure 8B). *NAC041* expression was reduced in accession 404 under osmotic and combined stress and induced in accession 395 under temperature stress. On the other hand, *NAC041* and *NAC084* were significantly overexpressed, 4.8-fold and 1.8-fold for temperature stress, respectively, and expression of *NAC084* increased 1.6-fold for combined stress in 395 accession. *NAC084* transcript was also significantly increased (1.7-fold) under combined stress in 404 accession and under mannitol stress (1.9-fold) in accession 411, while *NAC041* showed a tendency to decrease in both tolerant accessions, with significant changes only in accession 404.

### 2.8. Protein Immmunodetection

**Figure 9:**
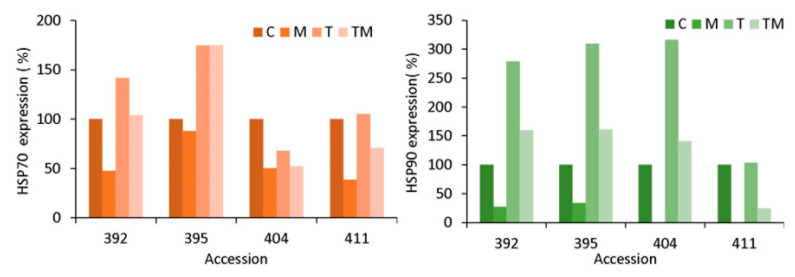
Immunodetection of heat shock proteins HSP70 and HSP90 in kale accessions 392, 395, 404 and 411 under osmotic (M), temperature (T) and combined stress (TM) compared to the corresponding controls (C). Data are expressed as % compared to the control (C = 100%).

Heat shock proteins HSP70 and HSP90 were analyzed by immunodetection (Western blot) and protein signals were semi-quantified and expressed as % compared to the control (C = 100 %) (Figure 9).

Representative Western blot images are presented in Figure S1. It is noticeable that more tolerant accessions 404 and 411 contained a higher level of HSPs in the control conditions compared to more sensitive accessions 392 and 395 (Figure S1A–C). As can be seen, HSP70 and HSP90 were downregulated in osmotic stress in all accessions, but particularly in accessions 392, 404 and 411. The smallest decrease in HSP70 under osmotic stress was in the 395 accession. HSP70 was markedly upregulated under temperature stress in accessions 392 and 395 (up to 140% and 180%, respectively), while it was downregulated or unchanged in accessions 404 and 411, respectively, compared to the control. Under combined stress, HSP70 was unchanged in the 392 accession, increasing up to 180% in the 395 accession and downregulated in accessions 404 and 411 (50% and 40% lower compared to the control). HSP90 was increased up to 300% in accessions 392, 395 and 404 under temperature stress, while it was unchanged in the 411 accession. A similar trend was observed under combined stress—HSP90 was increased slightly in accessions 392, 395 and 404, but decreased significantly in the 411 accession.

### 2.9. Stress Response: Interactions of Accession and Stress Treatment

Using the two-way ANOVA method, we analyzed the impact of accession (A), stress treatment (T) and their interaction (A×T) on the stress response of the selected kale accessions (Table 2). As shown, the majority of the measured parameters was highly influenced by the interaction of accession and treatment.

On the other hand, variations in total phenolics were strongly influenced by accession and to a lower level by the stress treatments, while H_2_O_2_ content was more influenced by the stress treatment than accession. Both photosynthetic parameters, PI_abs_ and F_v_/F_m_, were influenced by the accession and stress treatment, but not their interaction. Proline and root growth were significantly affected by treatment, accession and their interactions. Transcription factor NAC041 was also influenced by accession and stress treatment, while NAC084 was only affected by the kale accession.

**Figure 10:**
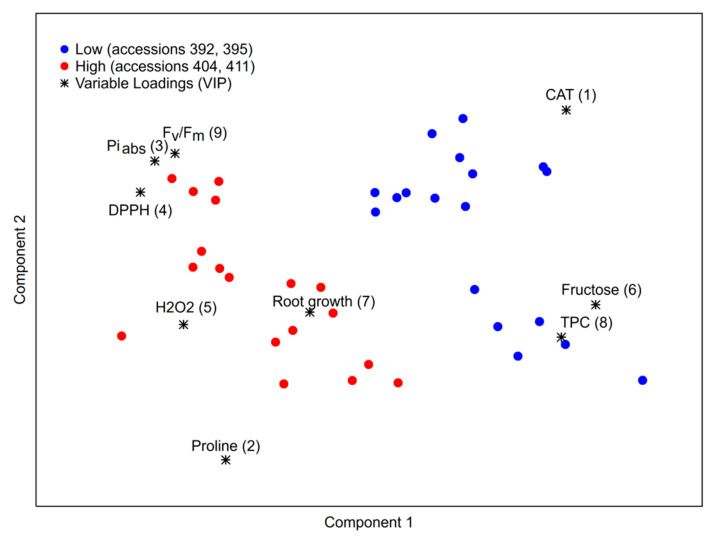
Multivariate analysis of parameters obtained for selected accessions 392, 395, 404, 411 under abiotic stress conditions (Table S2).

Figure 10 shows the multivariate analysis of all parameters obtained for four selected accessions under abiotic stress treatments. As can be seen, high-tolerant (red dots) and low-tolerant (blue dots) accessions were grouped separately based on the measured parameters. The most reliable markers for distinguishing between tolerant and sensitive kale accessions are CAT activity and proline content. Other important parameters are: photosynthetic parameters PI_abs_ and F_v_/F_m_, antioxidant activity measured by DPPH, H_2_O_2_, root growth, fructose and total phenolics (TPC). These variables were selected and presented in the figure according to the variable loading number (VIP) higher than the value 1 (Table S2). In addition, flavonoids, glucosinolates and transcript level of NAC041 also contributed significantly (VIP approximately 0.9; Table S2). DREB2A and HSFA7 transcript levels and MDA contributed to tolerance characterized by low VIP (below 0.5).

## 3. Discussion

Abiotic stress factors, such as high temperatures and drought associated with current climate changes, are affecting plant growth and crop yields for many agricultural crops, including Brassicaceae. Although many experimental designs examine individual stress factors, the situation in nature is more complex and plants are often exposed to complex stress factors in their habitat. To elucidate tolerance mechanisms in *Brassica*, we studied their responses to single elevated temperatures and osmotic stresses as well as to combined stress (elevated temperature + osmotic stress). Our previous work, including comparative studies of stress responses of *Brassica* among different species/varieties (Chinese cabbage (*Brassica rapa* ssp. *pekinensis*), white cabbage (*Brassica oleracea* var. *capitata*) and kale (*Brassica olerace* var. *acephala*)), showed that kale was the most tolerant white cabbage that was moderately tolerant and Chinese cabbage most sensitive to drought and salt stress [11,12,16]. We showed that bioactive molecules, such as phenolic compounds [16] and plant hormones [11,12,17], are highly involved in the mechanisms of abiotic stress tolerance in the studied *Brassica* plants. However, the mechanisms of Brassica tolerance to abiotic stresses involve complex networks of many players, such as reactive oxygen species (ROS), plant hormones, specialized metabolites, osmolytes, enzymatic and non-enzymatic antioxidants, etc., and are still far from being fully understood [18,19,20]. The stress response is highly dependent on the particular plant species/varieties. To better understand the mechanisms of stress response, we focused on kale (*Brassica oleracea* var. *acephala*) and screened 33 kale ecotypes or accessions for heat, osmotic and combined stress. Furthermore, we selected four accessions with different stress responses (392 and 395 as more sensitive and 404 and 411 as more tolerant) and used them for a more detailed analysis of stress response mechanisms. Among the stress treatments, the highest stress intensity, i.e., the most severe growth inhibition of seedlings, was associated with osmotic stress and combined stress, whereas temperature stress alone caused less noticeable changes compared with controls (Figure 1 and Figure 2). The results are consistent with data published on *Brassica napus* exposed to drought, heat and combined stress [21]. Among the selected accessions (392, 395, 404 and 411) (Figure 2), it is evident that each of them responded differently to individual and combined stress. Thus, the 392 accession was the most sensitive, while 411 seems to be the most tolerant to all stress conditions. Accession 395 appears to be more tolerant to combined stress than the other accessions, while 404 is very tolerant to osmotic stress.

### 3.1. Stress Markers

Seedling biomass production, root growth and proline content were selected as strong and reliable stress markers, showing stress status of the ecotypes studied, and were used to screen 33 kale ecotypes for tolerance (Figure 1 and Figure 2). A decrease in biomass accumulation, inhibition of root growth, decrease in water content and an increase in proline content were associated with higher stress status, particularly under osmotic and combined stress. This is in agreement with previous results obtained in *Brassica* crops treated by drought and salinity stress [11,12,17]. Plants under stress actively suppress their growth in order to survive under adverse conditions. Although beneficial for plant survival, active growth inhibition is often undesirable for crop productivity [22]. The effect of stress on plant growth can be measured as a decrease in plant growth rate or as a decrease in biomass accumulation. There is a strong positive correlation between biomass accumulation and root growth and stress tolerance under our experimental setup, showing that more tolerant accessions had lower suppression of biomass accumulation under stress conditions compared to the corresponding controls (Figure 1 and Figure 2). It was particularly observable in 404 and 411 accessions that were selected as tolerant ones for further examination of tolerance mechanisms. The correlation between proline accumulation and stress tolerance is controversial [23]. Comparative studies between *Brassica* species/varieties under drought and salt stress indicate that a higher increase in proline content is associated with more sensitive species/varieties [11,13], which is in agreement with the results obtained here, while a positive correlation between proline accumulation and stress tolerance was found in a comparative study of different *Brassica juncea* cultivars [24] and in rapeseed (*B. napus*) [25] subjected to abiotic stress. Another interesting observation is that more tolerant accessions had higher basal proline content than sensitive accessions under the same control conditions, which is consistent with previously reported data in *Brassica* plants [11,16].

Additional stress markers analyzed in selected accessions (392, 395, 404, 411) that confirmed stress status were H_2_O_2_, CAT, lipid peroxidation (MDA) and GSH content (Figure 4). As can be seen, H_2_O_2_ level was strongly dependent on accession. It was generally elevated in all accessions under stress treatments, except for 395. Among the various ROS usually generated under stress conditions, freely diffusible and relatively long-lived H_2_O_2_ can cause certain damage to cell structures, but also act as signaling molecules that turn on stress-response mechanisms [26,27,28]. These mechanisms can then activate a network of processes that enhance tolerance to various abiotic stressors. Under the stress conditions prevailing in our experiment, the more tolerant accessions 404 and 411 accumulated higher H_2_O_2_ concentrations compared to the more sensitive ones (392 and especially 395) (Figure 4A). This was associated with lower CAT activity, lower proline content and lower concentration of MDA, as a marker of lipid peroxidation, suggesting lower stress intensity suffered by accessions 404 and 411. These observations are in agreement with publications showing that exogenous application of H_2_O_2_ to rapeseed (*B. napus*) attenuates the effects of drought stress by mediating hormonal status and oxidative response [29,30]. Mechanism of activity of H_2_O_2_ as a signaling molecule seems to be complex and it is still unclear. It was shown that H_2_O_2_ affects the expression of genes that are involved in plant responses to diverse environmental stresses. Stress-induced H_2_O_2_ acts as signal in complex cross-talk of plant hormones ABA and auxin in response to salinity and drought [27] by disrupting the interaction between tryptophan synthase β subunit 1 (TSB1) and β-glucosidase 1 (BG1) that are involved in ABA and auxin homeostasis. Furthermore, a recent study showed that H_2_O_2_ activates plant cold responses by sulfenylating cytosolic enolase2 (ENO2) and promoting its oligomerization, leading to enhanced nuclear translocation and transcriptional activation of C-repeat/DRE binding factor1 (CBF1) [28].

### 3.2. Photosynthetic Performance in Stress

Photosynthesis is a process that is highly influenced by abiotic stresses and impaired photosynthesis has negative effects on plant growth, biomass production and yield. Two commonly used photosynthesis parameters that serve as stress indicators in plants are the performance index (PI_abs_) and the maximum quantum yield of PS II (F_v_/F_m_), both of which are determined by measuring chlorophyll a fluorescence that originates almost exclusively from PS II [31]. The values of PI_abs_ parameter confirmed a much higher responsiveness compared to F_v_/F_m_ (Figure 3), which is consistent with published data obtained on different cultivars of white and red cabbages [32]. Our results showed that PI_abs_ and F_v_/F_m_ were decreased significantly more in accessions 392 and 395, particularly under osmotic stress compared to the corresponding controls, than in more tolerant accessions 404 and 411 (Figure 3). This observation is in agreement with stress intensity level, measured as biomass accumulation, root growth and proline content. Moreover, our results correspond to data observed for *Brassica* species/varieties under drought stress [11]. Under our experimental conditions, temperature stress caused a significant decrease in photosynthetic performance only in the 395 accession, indicating a relative tolerance of this trait in kale to heat stress. Rodríguez et al. [33] compared white cabbage and kale under temperature stress and reported that low temperatures had a greater effect on *B. oleracea* physiology than high temperatures. A similar response, namely a greater decrease in chlorophyll content at low than at high temperatures, was observed in the study by Soengas et al. [34]. On the other hand, photosynthesis and respiration rates and the maximum quantum yield of photosystem II in developing seeds of *B. napus* were inhibited by heat stress that severely impairs yield and oil content [35]. Interestingly, our study showed that combined stress (high temperature + osmotic stress) caused fewer disturbances in photosynthetic performance than osmotic stress alone and did not cause a significant decrease in PI_abs_ or F_v_/F_m_ parameters. In the case of *B. napus*, reductions in net photosynthetic assimilation rate were caused by combinations of heat and drought (heat + drought) treatments, by drought alone and, to a lesser extent, by heat alone [36]. Our results (Table 2) as well as the published data cited above suggest that the effect of abiotic stress on photosynthetic parameters depends on both species/varieties and stress treatment.

### 3.3. Specialized Metabolite Accumulation under Stress Conditions

Specialized metabolites, such as phenolics, are generally recognized as molecules involved in stress protection in plants [37]. Under our experimental conditions, total phenols did not change significantly compared with the control, except for accession 411 under osmotic and combined stress. Total flavonoids tended to decrease under all stress condition in all examined accessions, particularly under osmotic and combined stress (Figure 5). Among selected accessions, 404 contained the highest content of flavonoids under both control and stress conditions and, consequently, showed the highest antioxidant activity.

Glucosinolates are specialized metabolites specific to Brassicaceae species. Their content was higher in accessions 395, 404 and 411 than in 392 under control conditions. In all accessions, the total content of glucosinolate tended to decrease under stress conditions, most markedly under osmotic and combined stress. Elevated temperature was found to increase glucosinolate concentrations in *B. rapa* while decreasing their content in *B. napus* [38]. Thus, fluctuations in glusosinolates under stress conditions depend on plant species, plant developmental stage, stress intensity as well as stress duration. The authors speculated that down-regulation of glucosinolate pathways under stress conditions may serve as a protective measure to conserve energy to ensure survival under adverse environmental conditions. Extensive studies on the Brassicaceae family showed a positive correlation between abiotic stress tolerance and glucosinolate content in broccoli, canola, radish sprouts and pakchoi [39].

### 3.4. Soluble Sugar Fluctuations under Stress Conditions

Sugars are the major building blocks for carbohydrate storage, but also serve as signaling molecules and protective compounds during abiotic stress exposure. Among the metabolites accumulated as osmoprotectants, numerous carbohydrates, including fructose, sucrose, trehalose, raffinose and fructans that are of high solubility, have been shown to accumulate in response to abiotic stresses [40]. Our data showed that total soluble sugars increased significantly in accession 392 under temperature and combined stress (Figure 5A). The individual sugars (trehalose, sucrose, glucose and fructose) were analyzed by HPLC (Figure 5B). Fructose content was increased significantly under osmotic and temperature stress in sensitive accessions (392 and 395), while it decreased in 404 or was not changed in 411. Glucose content was decreased, particularly under osmotic and combined stress (392, 395, 404), or remained unchanged (411) compared to the controls. Trehalose+sucrose decreased significantly under osmotic stress and increased significantly under temperature stress in all accessions. Based on our results, there is no general conclusion about fluctuations in individual energy source sugars and stress tolerance. Fluctuations in individual sugars under abiotic stress in kale depend on plant ecotype, stress factors, intensity and duration of stress, which is in agreement with results obtained on canola (*B. napus*) [41].

It is interesting that accessions 392 and 395 contained higher levels of fructans compared to accessions 404 and 411 in the control conditions. Osmotic and combined stresses caused a significant decrease in fructans in 392 and 395 while heat did not change it. On the other hand, fructans content was significantly increased in 404 under all stress conditions. Fructans are the non-structural carbohydrates, possessing other physiological functions than carbon reserve [42]. Fructans have been reported to have a protecting role in plants against water deficit caused by drought and osmotic stress. Plants synthesize fructans in order to osmoregulate the cellular flux, therefore, reducing the membrane damage. In addition, fructans are recognized as excellent scavengers of ROS [43]. Our results suggest that one of the mechanisms of abiotic stress tolerance in kale is an increase in fructans content.

### 3.5. Stress-Related Transcription Factor Gene Expression and Heat Shock Protein Accumulation

Plant stress response to unfavorable environmental conditions includes developmental, physiological and biochemical changes, guided by stress-related transcription factors. Drought and heat stress are initially sensed by membrane-localized stress receptors, intracellularly relayed through secondary messengers, especially calcium ions that activate signaling pathways through mitogen-activated (MAPK) and calcium-dependent (CDPK) protein kinases. Several transcription factors, such as *HSFs*, *DREB* and *NAC* (NAM, ATAF and CUC domain), are upregulated by kinases (MAPKs and CDPKs) and induce synthesis of stress-responsive proteins that, in turn, ensure plant survival in stressful conditions. Tolerance or susceptibility to stress is, therefore, dependent on the ability of the plant to express a set of genes whose expression is often regulated by *HSF*, *DREB* and *NAC* transcription factors [44,45,46]. These transcription factors (TFs) regulate the expression of chaperones, such as heat shock proteins (HSPs), detoxification of reactive oxygen species (ROS), expression of antioxidant enzymes (ascorbate peroxidase, APX, catalase, CAT) and expression of other genes involved in signal transduction and regulatory pathways. They play critical roles, not only in heat stress response, but also in other abiotic stresses [19,32] and their transcriptional reprogramming or genetic manipulation is considered a valuable tool in engineering stress-tolerant varieties of crop plants [19,44,47]. Zhu et al. reported that the most *B. napus* HSFs were induced under heat as well as drought stress, suggesting their role in multiple abiotic stress responses in canola [46]. This is in agreement with our results, showing that transcript level of *HSFs* was significantly increased under heat stress, but also under osmotic stress in the most sensitive accession 392 and under combined stress in all, so the 411 accession was the most resistant (Figure 8). Our results suggest that more sensitive accessions increased expression of HSFs more prominently compared to the more tolerant accessions.

Unlike moderate increases in *HSFA2* and *HSFA7*, *DREB2*, a transcription factor that mediates high salinity, dehydration- and heat-stress-inducible transcription [9,48], had the highest induction under combined stress in all except the sensitive 392 accession, from which it can be concluded that exposure to drought- and heat-stress-tolerant cultivars has modest induction of *HSFA2* and *HSFA7* and more noticeable induction of DREB2A in comparison to more sensitive accessions.

There are multiple regulatory interactions between *HSFs* and HSPs [49]. Consequently, accumulation of HSPs was higher in sensitive accessions (392 and 395) compared to more tolerant (404 and 411) (Figure 9 and Figure S1). Earlier, HSPs were believed to produce under heat stress, as the name indicates, but now it is established that these biomolecules are produced in response to various biotic and abiotic stresses [50]. HSP90 works in association with HSP70 as a major part of chaperone complexes. While the major role of HSPs is protein folding, they also act as the key component in signal-transduction networks, cell-cycle control, protein trafficking, etc. A study on chickpea HSP70 reported that HSPs were first downregulated in the early stage of growth in drought-tolerant cultivars, which is in agreement with our results. In contrast, HSPs were abundant in drought-sensitive cultivars, which indicated that HSPs responded to drought not only in the specific genotypes but, also, during the developmental stage [51].

The role of *NAC* TFs in abiotic stress tolerance is well documented [19,52,53]. A number of *NAC* transcription factors has already been functionally characterized in model plants, demonstrating their role in abiotic stress. In response to heat and drought stress, many *NAC* transcription factors in *B. rapa* are repressed [52,54], which is consistent with our results, showing downregulation of *NAC041* under osmotic and combined stress in the kale accessions, except for 395, which had a significantly reduced basal *NAC041* expression level. On the other hand, *NAC084* showed a higher basal expression level in tolerant kale accessions and a tendency to overexpress under stress conditions, particularly in more tolerant accessions (Figure 7 and Figure 8). Overall, our results suggest that the higher basal expression of *NAC04*, *NAC084* and *DREB2A* and the change in *HSFA2* and *HSFA7* can be used as valuable markers in screening heat- and drought-tolerant kale cultivars.

## 4. Materials and Methods

### 4.1. Plant Material

Seeds of 33 kale accessions (*Brassica oleracea* var. *acephala*) were collected from farmers along the Croatian coast and islands in 2018 and 2019, including four accessions from Bosnia and Herzegovina (385, 393, 395 and 413) (Table 1). None of the collected accessions are used for commercial purposes and they are reproduced and maintained by local farmers usually as a part of family inheritance. The collected seeds of all accessions were regenerated in the season 2019/2020 at the Institute of Agriculture and Tourism and used for this experiment.

### 4.2. Plant Growth and Abiotic Stress Treatments

The level of abiotic stress tolerance to osmotic stress, high-temperature stress and combined stress (osmotic + temperature stress) of 33 kale accessions was determined using the root-growth bioassay described earlier [17] with slight modifications. After sterilization, seeds were placed onto 1% agar plates and stratified at 4 °C for 5 days. Plates were then moved to a growth chamber (PHC Corporation, Tokyo, Japan), positioned vertically, at 22 °C, 16 h of light at 150 µmol m^−2^ s^−1^/8 h of darkness. One-day-old seedlings with approximately 1 cm long roots were subjected to stress as follows: osmotic (M)—seedlings transferred to 1% agar plates containing 0.3 M mannitol and incubated at 22 °C; elevated temperature (T)—seedlings transferred to 1% agar plates under the following temperature cycle: 24 °C for 10 h during the night period (10 p.m.–8 a.m.), 28 °C for 4 h (8:00 a.m.–12), 38 °C for 4.5 h (12–4:30 p.m.), 28 °C 5.5 h (4.30 p.m.–10 p.m.); combined osmotic + elevated temperature (TM)—seedlings transferred to 1% agar plates containing 0.3 M mannitol under the temperature cycle described above for T treatment. Seedlings transferred to 1% agar plates and incubated at 22 °C were used as control (C). Treatments were performed for 3 days, in growth chambers at a 16/8 photoperiod and a light intensity of 150 µmol m^−2^ s^−1^. Before harvesting the seedlings, the plates with seedlings were scanned for root analysis and the seedlings were weighed for biomass determination. The roots were analyzed using the ImageJ program. After photosynthetic measurements, the seedlings were quickly frozen in liquid nitrogen and stored at −80 °C. Plant material used for analyses of sugars, specialized metabolites, proline, malondialdehyde (MDA) and protein immunodetection was freeze dried before analyses.

### 4.3. Photosynthetic Measurements

Photosynthesis measurements were performed using six individual seedlings per treatment and control in vivo. Fast chlorophyll kinetics was measured on dark-adapted (30 min of light deprivation) cotyledons using Fluorpen (Photon Systems Instruments). The cotyledons were flattened on wet filter paper to make the surface area large enough for measurement. To induce chlorophyll fluorescence transients (OJIP), they were exposed to a saturation pulse (455 nm, max. 3000 μmol m^−2^ s^−1^) and fluorescence intensity was measured from 50 μs after the pulse (F_0_) until 1 s (F_m_). From these measurements the JIP test was performed to calculate related parameters [55] from which photosynthetic performance index (PI_abs_) and the maximum quantum yield of PSII (F_v_/F_m_) were used.

### 4.4. Biochemical Stress Markers

H_2_O_2_ analysis was performed using plant material stored at −80 °C, while other parameters were determined using previously lyophilized plant material. Extracts for determination of H_2_O_2_ content were prepared by homogenization of frozen samples (−80 °C) with glass and ceramic beads in 70% ethanol. The solution was centrifuged at 5000× *g* for 20 min at 4 °C. H_2_O_2_ content was determined spectrophotometrically using FOX reagent (0.25 mM ammonium iron (II) sulfate, 124 μM xylenol orange, 99 mM sorbitol) [56]. Sample extracts were mixed with FOX reagent in the ratio of 1:10 (extract:reagent), incubated for 15 min at RT and absorbance was measured at 560 nm. H_2_O_2_ content was calculated using a standard curve of known H_2_O_2_ concentrations and expressed as µg per fresh weight (µg g^−1^ FW).

For proline determination, lyophilized tissue was homogenized in 70% ethanol using glass beads and incubated for 30 min at 4 °C. The solution was centrifuged for 10 min at 16,000× *g* at 4 °C. Free proline was determined spectrophotometrically using acid ninhydrin (1% (*w*/*v*) ninhydrin, 60% (*v*/*v*) glacial acetic acid, 20% (*v*/*v*) 96% ethanol) as described [16]. The mixture was incubated at 95 °C for 20 min and absorbance was measured at 520 nm. Proline content was calculated using a standard curve of known L-proline concentrations and expressed as µmol L-proline per dry weight (µmol g^−1^ DW).

Protein extracts were prepared by grinding lyophilized tissue in cold potassium phosphate buffer (0.1 M, pH 7.0) with the addition of polyvinylpolypyrrolidone. After centrifugation at 16,000× *g* for 20 min at 4 °C, protein concentration in the supernatant was measured using the Bradford assay [57]. The supernatants were stored at −20 °C and used for further analyses.

Catalase (CAT) activity was measured using protein extracts according to the method of Aebi [58]. Protein extracts were added to reaction buffer (0.1 M potassium phosphate buffer, 10 mM H_2_O_2_, pH 7.0) in a ratio of 1:40 (extract:reaction buffer) and the change in absorbance at 240 nm was measured every 10 s for 1 min. Catalase activity was calculated using the extinction coefficient of 40 mM^−1^ cm^−1^. Results were expressed as nmol of decomposed H_2_O_2_ per minute per milligram of total soluble proteins (nmol min^−1^ mg^−1^ proteins).

Lipid peroxidation was determined spectrophotometrically in protein extracts as described by Draper and Hadley [59]. Thawed protein extracts were added to 20% (*w*/*v*) trichloroacetic acid (TCA) and separately to TBA/TCA reagent (0.3% (*w*/*v*) thiobarbituric acid prepared in 20% TCA) in a ratio of 1:3 (extract:reagent). The mixtures were incubated for 30 min at 95 °C, centrifuged at 15,000× *g* for 10 min at 4 °C and the precipitate was discarded. Absorbance was measured at 532 and 600 nm and MDA content was calculated using the extinction coefficient of 155 mM^−1^ cm^−1^. Results were expressed as nmol MDA per protein (nmol mg^−1^ proteins).

### 4.5. Protein Immmunodetection

Proteins were extracted in Tris-HCl buffer, pH 8.0 [60], quantified as described above and separated using vertical sodium dodecyl sulfate polyacrylamide gel electrophoresis (SDS-PAGE). A discontinuous Tris-Gly buffer (pH 8.3) system was used (4% stacking gel, 12% resolving gel). The proteins were denatured using Laemmli buffer [61] at 96 °C for 5 min and 6 μg of protein per sample was loaded onto the gel. The separated proteins were electrotransferred to nitrocellulose membrane in transfer buffer (0.335 % Tris, 1.44% Glycine, 10% methanol) at 60 V for 60 min. Before blocking, the efficiency of the transfer was checked by incubating the membrane in Ponceau rouge solution (0.05% Ponceau rouge). After washing in TBS buffer (0.24% Tris, 0.88% NaCl, pH 7.5), the membrane was blocked with 5% milk solution in TTBS buffer (TBS buffer, 0.05% Tween 20) for 1 h. The membrane was then incubated overnight at 4 °C in anti-HSP70 (Agrisera, AS08371) diluted 1:1000 in TTBS buffer containing 3% milk or anti-HSP90 (Agrisera, AS08346) diluted 1:3000 in TBS buffer. The membrane was washed in blocking solution, incubated with secondary antibody (anti-rabbit IgG-horseradish peroxidase (HRP) diluted 1:5000 in TTBS with 5% milk), 60 min at room temperature and washed in TTBS. Signals were detected using chemiluminescent HRP substrate and the intensity of the bands was quantified using ImageJ.

### 4.6. Specialized Metabolites, Antioxidant Capacity and GSH

Approximately 10 mg of freeze-dried plant tissue was homogenized with 500 μL of aqueous methanol (80:20, methanol:water, *v*/*v*). The homogenate was incubated in an ultrasonic bath (Sonorex, Bandelin) at 20 °C for 30 min and then centrifuged at 15,000× *g* for 5 min. The extracts were stored at −80 °C and used for the following analyses.

Total phenolics were assayed using the Folin–Ciocalteu method [62]. Briefly, 300 μL of 1.88 M Na_2_CO_3_ was added to 1580 μL of dH_2_O, 20 μL of the extract and 100 μL of the Folin–Ciocalteu reagent. The reaction mixture was mixed and incubated in a dry-block heater at 45 °C for 1 h. The absorbance was measured at 765 nm and total phenols were expressed as equivalents of gallic acid per dry weight (mg GAE g^−1^ DW).

Total flavonoids were determined by the AlCl_3_ method [63], with minor modifications. The reaction mixture consisted of 100 μL of the extract, 20 μL 10% (*w*/*v*) AlCl_3_, 500 μL 1 M potassium acetate and 380 μL dH_2_O. The absorbance of the mixture was measured at 420 nm after 30 min of incubation at room temperature (25 °C). Results were expressed as quercetin equivalents per dry weight (mg Q g^−1^ DW).

Total glucosinolates were measured as previously described [64], with some modifications. In short, 30 μL of the extract was mixed with 900 μL of 2 mM Na2PdCl4. After 30 min of incubation at room temperature (25 °C), the absorbance was measured at 425 nm and results were expressed as sinigrin equivalents per dry weight (mg SEQ g^−1^ DW).

Total antioxidant capacity was evaluated using the DPPH radical scavenging capacity assay [65]. The extract (50 μL) was added to 950 μL of 0.1 mM DPPH reagent prepared in 96% ethanol, mixed and incubated for 30 min at room temperature (25 °C). The absorbance was read at 517 nm and results were expressed as Trolox equivalents per dry weight (μmol TE g^−1^ DW).

GSH was measured according to Malar et al. [66]. Briefly, 20 μL of the extract was added to 940 μL of potassium phosphate buffer (0.1 M, pH 7.0) and 40 μL of 0.01 M 5,5 -dithio-bis-(2-nitrobenzoic acid) previously prepared in potassium phosphate buffer (0.1 M, pH 7.0). The mixture was incubated at room temperature for 5 min and absorbance was measured at 412 nm. GSH content was calculated using a standard curve of known GSH concentrations and expressed as mmol GSH per dry weight (mmol g^−1^ DW).

### 4.7. Sugar Analysis

Total sugars were determined by the anthrone method [67]. To 1 mL of 2% (*w*/*v*) anthrone prepared in 71.33% (*v*/*v*) H_2_SO_4_, 200 μL of the extract was added. The reaction mixture was incubated at 90 °C for 5 min. After cooling to room temperature, absorbance was measured at 620 nm. Results were expressed as sucrose equivalents per dry weight (mg sucrose g^−1^ DW).

For more detailed sugar analysis, the lyophilized plant tissue (approximately 150 mg) was homogenized with 2.4 mm metal beads (Omni kit 19-670, Kennesaw, GA, USA) for 1 min at 5 m s-1 in 3 mL of 80% methanol in water using a bead mill (Omni Bead Ruptor Elite, Kennesaw, GA, USA). The homogenates were left to macerate for 1 h on a rotator (Biosan RS-60, Riga, Latvia) and subsequently centrifuged for 5 min at 5000× *g*. The extracts were filtered through a 0.22 µm nylon filter prior to analysis. The analysis of fructans, trehalose, sucrose, glucose and fructose content was carried out using an HPLC system consisting of a system controller (Shimadzu CBM-40, Kyoto, Japan), a degassing unit (Shimadzu DGU-405, Kyoto, Japan), a solvent delivery unit (Shimadzu LC-20Ai, Kyoto, Japan), an autosampler (Shimadzu SIL-20AC, Kyoto, Japan), column oven (Shimadzu CTO-40S, Kyoto, Japan) and a refractive index detector (Shimadzu RID-20A, Kyoto, Japan). Chromatographic separation was achieved by injecting 10 µL of the sample on a 300 × 8 mm, 9 µm particle size, calcium cation exchange column (Dr. Maisch ReproGel Ca, Ammerbuch, Germany) held at 80 °C using deionized water as the mobile phase (0.6 mL min-1, isocratic elution). Retention times and peak areas of the investigated sugars were compared to analytical standards for identification and quantification, respectively. The retention time of sucrose and trehalose was identical and, therefore, the result was expressed as a sum of both sugars. Linear calibration curves were obtained with serial dilutions of 0.1, 0.5, 1.0, 5.0 and 10.0 g L-1 of inulin (standard for fructans) (y = 124.059x + 651.0289, coefficient of determination, R2 = 0.99999), sucrose (y = 135.759x − 1629.46, coefficient of determination, R2 = 0.99999), glucose (y = 138.942x − 1427.89, coefficient of determination, R2 = 0.9999) and fructose (y = 133.871x − 2662.79, coefficient of determination, R2 = 0.9999).

### 4.8. Quantitative Real-Time PCR (RT-PCR) Analysis

Total RNA was isolated from frozen 3-day old kale seedlings using the MagMAx Plant RNA Isolation Kit (Thermo Scientific, Waltham, MA, USA), according to the manufacturer’s instructions. One biological replicate was composed of 10 seedlings grown either under control or stress conditions (mannitol, temperature treated or combined stress treated). After extraction RNA was quantified using NanoDropTM 1000 Spectrophotometer (Thermo Scientific). Isolated RNA (1 µg) was reverse transcribed in a total reaction volume of 20 µL using 200 U of RevertAid H Minus Reverse Transcriptase (Thermo Scientific), 1× Reaction Buffer (Thermo Scientific), 20 U of RiboLock RNase inhibitor (Thermo Scientific), 1 mM dNTPs (Sigma-Aldrich) and 2.5 µM Oligo(dT)18 primer (Thermo Scientific). For cDNA synthesis, the reactions were incubated at 65 °C for 5 min, at 42 °C for 1 h and at 70 °C for 15 min, followed by a five-fold dilution with water. Genomic DNA (gDNA) was extracted with CTAB [68]. All primers used (Table 3) were designed based on gene sequences of *Brassica rapa* ssp. *pekinensis* and *Brassica oleracea* ssp. *oleracea* and checked by standard PCR on gDNA and cDNA of kale cultivars. Standard PCR reactions contained 1× EmeraldAmp^®^ GT PCR Master Mix (Takara Bio Inc., Kusatsu, Japan), 300 nM forward and reverse qB-OGIO primer and 2 µL (20 ng) of cDNA or genomic DNA in a total volume of 25 µL. PCR was performed in a thermocycler (Eppendorf Mastercycler, Hamburg, Germany) with the initial denaturation step set at 95 °C for 2 min, followed by 35 cycles of denaturation at 95 °C for 30 s, annealing at 58–60 °C for 30 s, extension at 72 °C for 1 min and a final extension step at 72 °C for 5 min. To check cDNA quality standard PCR reaction with qB-OGIO primers was performed. qB-OGIO forward and reverse primers are complementary to different exons and in case of undesirable gDNA presence in cDNA sample two fragments are synthesized indicating an unsuitable sample for qPCR. Quantitative RT-PCR was performed in duplicate on the MIC platform (Bio molecular Systems). A total reaction volume of 15 µL contained 1× GoTaq^®^ qPCR Master Mix reagent (Promega, Madison, WI, USA), 133 nM of forward and reverse primers (Table 1) and 20 ng (2 µL) cDNA. The run profile of the PCR reaction was as follows: 95 °C for 5 min followed by 40 cycles of 95 °C for 5 s and 58 °C for 10 s. Melting curves were generated from 40 °C to 95 °C at a ramp speed 0.3 °C s^−1^ to check for specific amplification. For normalization, reference genes OGIO (Bra028284) and PUX (Bra026205) were used as internal controls [69]. Relative gene expression was calculated according to Livak and Schmittgen [70].

### 4.9. Statistical Analysis

The experiments were performed with three biological replicates unless otherwise stated. Each plate represented one biological replicate consisted of 15 seedlings grown under the same conditions. To visualize similarities in the level of abiotic stress tolerance among 33 different kale accessions, heatmap and cluster analyses were performed on root length, biomass and water content and proline level data, using R 4.1.1. Software and “ComplexHeatmap” package [71]. The dendrogram was constructed using Euclidean distance.

Further analyses were performed on selected tolerant (404 and 411) and sensitive (392 and 395) kale accessions including all measured parameters except biomass and water content of seedlings as well as protein immunodetection. Two-way analysis was used to determine the influence of kale accession and stress treatment and one-way ANOVA followed by LSD test to find whether there were differences between the control and treatment groups of a particular kale accession. Results were considered significantly different at *p* ≤ 0.05.

To further reveal the differences between accessions with lower or higher stress tolerance a partial least-squares–discriminant analysis (PLS-DA) model was developed based on the obtained results. To prevent overfitting of the developed model a cross-validation step was performed using the leave-one-out cross-validation method [72]. For each parameter a variable importance in projection (VIP) value was calculated and only those parameters with a value higher than one were deemed important. Statistical analyses were performed using TIBCO Statistica 13.5.0.17 software package (TIBCO Software Inc., New York, NY, USA).

## 5. Conclusions

To elucidate the mechanisms of abiotic stress tolerance, 33 kale accessions (*B. oleracea* var. *acephala*) were evaluated for individual (osmotic and elevated temperature stress) and combined stress (osmotic+temperature). Growth performance (root growth and biomass), water content and proline content, as reliable stress markers, were used to select accessions with different stress tolerance. The most sensitive accession to all stress factors was 392. Accession 395 was quite sensitive to heat and mannitol but showed better tolerance to combined stress. More tolerant accession, especially to osmotic stress, was 404 and the most tolerant accession to all stress conditions was 411. The more tolerant accessions had higher basal content of proline, total soluble sugars, glucosinolates, heat shock proteins and higher transcript level of *NAC* and *DREB* transcription factors. On the other hand, sensitive accessions were characterized by a high basal content of fructans. Among stress conditions, mannitol and combined stress caused more prominent changes in morphological and biochemical parameters compared to high-temperature stress. Under stress conditions, the most sensitive accession, 392, was characterized by a significant decrease in biomass accumulation, root growth, photosynthesis performance, fructans content, particularly under osmotic and combined stress, as well as a significant increase in *HSF* transcript expression and HSP accumulation under heat stress and a significant decrease in *NAC* expressions under all examined stress conditions. On the other hand, the most tolerant accession, 411, experienced the lowest changes in all analyzed parameters compared to other accessions. Based on multivariate analysis of all measured parameters for selected kale accessions under abiotic stress treatments, accessions 392 and 395 were distinguished as low tolerant from accessions 404 and 411, which appeared to be more tolerant. The most informative variables in tolerance definition were photosynthetic parameters PI_abs_ and F_v_/F_m_, antioxidant activity, H_2_O_2_, proline, root growth, CAT, fructose and total phenolics.

## Figures and Tables

**Table 1:** List of kale (*B. oleracea* var. *acephala*) accessions used in tolerance evaluation. Kale accessions are part of the seed collection of Institute for Agriculture and Tourism Poreč.

Acc.	City	Country	Coordinates	
202	Kaštelir	Croatia	45°18′21.9708″ N	13°41′11.7018″ E
206	Labinci	Croatia	45°17′38.3244″ N	13°41′12.6636″ E
379	Fuškulin	Croatia	45°10′57.3492″ N	13°38′32.5608″ E
380	Island Iž 2.	Croatia	44°0′56.74″ N	15°8′21.19″ E
381	Preko, island Ugljan	Croatia	44°4′45.5916″ N	15°11′14.5212″ E
383	Vrgorac	Croatia	43°12′4.3806″ N	17°23′16.0326″ E
384	Konavle, Pavlje Brdo	Croatia	42°30′55.0296″ N	18°20′50.5494″ E
385	Vitina, Mostar	Bosnia and Herzegovina	43°14′33.5178″ N	17°28′29.3694″ E
386	Vrgorac	Croatia	43°11′28.1904″ N	17°21′53.643″ E
387	Ponikve, Pelješac	Croatia	42°50′52.983″ N	17°37′36.786″ E
388	Topići, Baška voda	Croatia	43°22′0.0912″ N	16°57′41.2698″ E
390	Island Lošinj	Croatia	44°34′53.097″ N	14°24′21.9018″ E
391	Ugljan, islland Ugljan	Croatia	44°8′0.1392″ N	15°6′8.7804″ E
392	Dubrovnik	Croatia	42°38′40.8264″ N	18°7′21.2592″ E
393	Mostar	Bosnia and Herzegovina	43°21′35.7186″ N	17°49′7.269″ E
394	Oključina, island Vis	Croatia	43°4′17.7348″ N	16°6′36.1296″ E
395	Mostar	Bosnia and Herzegovina	43°20′25.2564″ N	17°43′28.1274″ E
396	Island Lošinj	Croatia	44°41′9.942″ N	14°22′12.864″ E
397	Vrgorac	Croatia	43°12′25.7106″ N	17°21′36.8496” E
398	Vrgorac, Prapatnice	Croatia	43°13′43.85″ N	17°20′59.03″ E
399	Čarsko polje, island Korčula	Croatia	42°55′59.8362″ N	16°56′6.0828″ E
401	Zavalatica, island Korčula	Croatia	42°55′7.0968″ N	16°56′9.4272″ E
402	Katuni	Croatia	43°27′45.39″ N	16°53′10.39″ E
403	Drinovci	Croatia	43°21′33.0696″ N	17°19′23.9874″ E
404	Blato na Cetini, Omiš	Croatia	43°28′34.87″ N	16°49′7.39″ E
405	Opuzen	Croatia	43°0′19.28″ N	17°33′34.69″ E
406	Babino polje, isl. Mljet	Croatia	42°44′22.63″ N	17°30′2.93″ E
408	Poreč-Pištan	Croatia	45°13′29.33″ N	13°37′39.76” E
411	Island Iž 1.	Croatia	44°2′37.248″ N	15°6′43.671″ E
413	Tomislav grad	Bosnia and Herzegovina	43°43′7.39″ N	17°13′25.07″ E
414	Srijane	Croatia	43°31′20.94″ N	16°41′14.45″ E
416	Vinjani donji	Croatia	43°26′28.23″ N	17°14′25.66″ E
423	Kreševo	Croatia	43°29′14.65″ N	16°52′35.42″ E

**Table 2:** Two-way ANOVA summary table represents effect of accession (A), treatment (T) or accession × treatment (A×T) on total phenols, total flavonoids, antioxidative capacity, total glucosinolates, MDA content, CAT activity, H_2_O_2_ content, glutathione content (GSH), photosynthetic parameters F_v_/F_m_ and PI_abs_, total sugars, proline content, root growth and transcription levels of *NAC041*, *NAC084*, *HSFA2*, *HSFA7* and *DREB2A* genes. Results were considered as non-significant (ns) at *p* > 0.05, as significant (*) at *p* < 0.05, as very significant (**) at *p* < 0.01 and as highly significant (***) at *p* < 0.001.

Parameter	Source	SS	Df	MS	F	*p*	Significance Level
Total phenols	A	61.02	3	20.34	14.07	0.00000	***
	T	12.80	3	4.27	2.95	0.03868	*
	A×T	18.82	9	2.09	1.45	0.18598	ns
Total flavonoids	A	9.42	3	3.14	42.69	0.00000	***
	T	34.45	3	11.48	156.08	0.00000	***
	A×T	5.52	9	0.61	8.34	0.00000	***
Antioxidative activity	A	76.93	3	25.64	13.92	0.00000	***
	T	73.82	3	24.61	13.35	0.00000	***
	A×T	51.94	9	5.77	3.13	0.00327	**
Total glucosinolates	A	183.15	3	61.05	30.19	0.00000	***
	T	1137.48	3	379.16	187.5	0.00000	***
	A×T	111.58	9	12.40	6.13	0.00000	***
MDA	A	4.23	3	1.41	4.12	0.01404	*
	T	32.87	3	10.96	31.99	0.00000	***
	A×T	9.58	9	1.06	3.11	0.00847	**
CAT activity	A	2561.07	3	853.69	71.86	0.00000	***
	T	742.76	3	247.59	20.84	0.00000	***
	A×T	545.15	9	60.57	5.10	0.00027	***
H_2_O_2_ content	A	8.28	3	2.76	3.14	0.03864	*
	T	17.37	3	5.79	6.59	0.00136	**
	A×T	14.83	9	1.65	1.88	0.09231	ns
GSH	A	0.85	3	0.28	10.45	0.00001	***
	T	1.87	3	0.62	22.99	0.00000	***
	A×T	0.99	9	0.11	4.06	0.00034	***
F_v_/F_m_	A	0.00	3	0.00	3.40	0.02667	*
	T	0.00	3	0.00	6.10	0.00156	**
	A×T	0.00	9	0.00	1.00	0.42690	ns
PI_abs_	A	4.56	3	1.52	7.68	0.00034	***
	T	3.51	3	1.17	5.92	0.00184	**
	A×T	1.40	9	0.16	0.79	0.62964	ns
Total sugars	A	35.56	3	11.85	5.45	0.00204	**
	T	25.22	3	8.41	3.87	0.01296	*
	A×T	44.17	9	4.91	2.26	0.02835	*
	A	643.29	3	214.43	38.88	0.00000	***
Proline content	T	2011.56	3	670.52	121.59	0.00000	***
	A×T	284.31	9	31.59	5.73	0.00003	***
	A	41.05	3	13.68	7.04	0.00015	***
Root growth	T	598.51	3	199.50	102.68	0.00000	***
	A×T	104.58	9	11.62	5.98	0.00000	***
*NAC041*	A	2.63	3	0.88	12.63	0.00000	***
	T	1.46	3	0.49	7.00	0.00031	***
	A×T	0.68	9	0.08	1.09	0.37708	ns
*NAC084*	A	19.42	3	6.47	15.64	0.00000	***
	T	0.79	3	0.26	0.64	0.59454	ns
	A×T	3.05	9	0.34	0.82	0.60024	ns
*HSFA2*	A	4363.49	3	1454.5	4.75	0.00424	**
	T	38,958.62	3	12,986.21	42.41	0.00000	***
	A×T	14,531.79	9	1614.64	5.27	0.00001	***
*HSFA7*	A	3390.37	3	1130.12	26.89	0.00000	***
	T	7988.41	3	2662.80	63.36	0.00000	***
	A×T	8587.73	9	954.19	22.70	0.00000	***
*DREB2A*	A	50.25	3	16.75	0.69	0.55866	ns
	T	1884.68	3	628.23	26.01	0.00000	***
	A×T	928.47	9	103.16	4.27	0.00015	***

**Table 3:** Primer sequences used for standard and quantitative real-time PCR analysis.

Gene	Primer Name	Sequence 5′->3′
*DREB2A*	qB-DREB2A-Fw	TTTGATGTTTCTGAGCTTCTTGG
Bra009112	qB-DREB2A-Rev	CATTGTCTCCCAGGCATTGG
*HSFA2*	qB-HSFA2-Fw	ATGAATGTGATGATGGAAGATGGT
Bra000557	qB-HSFA2-Rev	CTGCCCCAATCCAACGGTG
*HSFA7A*	qB-HSFA7A-Fw	TCTGAGACAGCAGCAACAAAC
Bra012828	qB-HSFA7A-Rev	CTGGAGTAGCTGATACAGAAAC
*NAC041*	qB-NAC041-Fw	CGAAGACGACAACAAGAGTGC
Bra021856	qB-NAC041-Rev	GAGTCACATTCAAATCGCAGC
*NAC084*	qB-NAC084-Fw	AGGAAGAAGACAGAGGAAACC
Bra006229	qB-NAC084-Rev	GCTGAGGTAGGAGGAGATG
*OGIO*	qB-OGIO-Fw	CAGTATCGTAGCTGAGGTAGC
Bra028284	qB-OGIO-Rev	AGAACGGAACACATACTTGACTC
*PUX*	qB-PUX-Fw	CAAACCCAAAGAGGTTGTTGC
Bra026205	qB-PUX-Rev	TCATGTCGTTGTCTTCCAAGG

## Data Availability

Not applicable.

## Supplementary Materials

The following supporting information can be downloaded at: https://www.mdpi.com/article/10.3390/ijms231911494/s1.
