# Supplementary material for: Mechanisms of Kale (Brassica oleracea var. acephala) Tolerance to Individual and Combined Stresses of Drought and Elevated Temperature
Source: Int J Mol Sci. 2022 Sep 29;23(19):11494. doi: 10.3390/ijms231911494 (PMC9570052; doi:10.3390/ijms231911494)
Supplement: Supplementary file 1 [file ijms-23-11494-s001.zip › Figure S1.pdf]

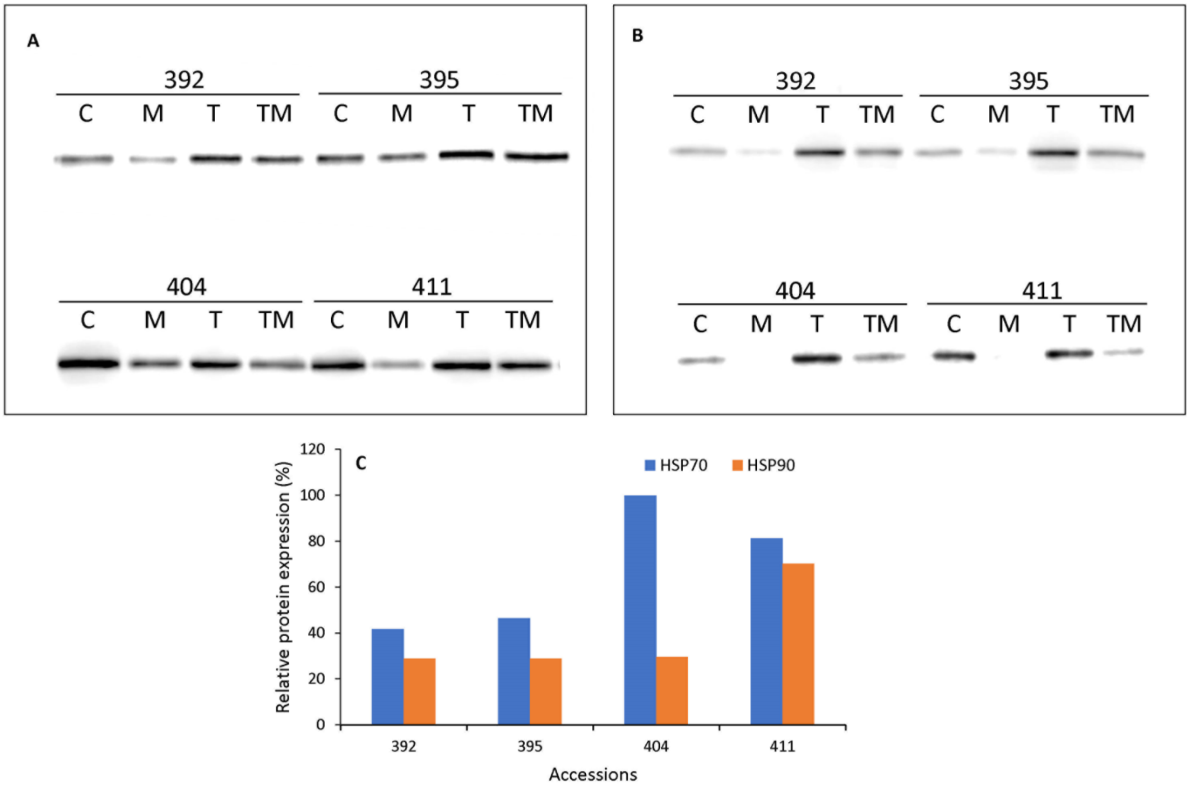

Figure S1: Representative Western blot image of heat shock proteins in accessions 392, 395, 404, and 411 under stress conditions (M-mannitol, T-elevated temperature, TM-combined stress, C-control: (A) HSP70; (B) HSP90; (C) relative protein expression of heat shock proteins HSP70 and HSP90 in accessions 392, 395, 404, 411 in control conditions (basal level).
